# Crystal Structure of Human Herpesvirus 6B Tegument Protein U14

**DOI:** 10.1371/journal.ppat.1005594

**Published:** 2016-05-06

**Authors:** Bochao Wang, Mitsuhiro Nishimura, Huamin Tang, Akiko Kawabata, Nora F. Mahmoud, Zahra Khanlari, Daizo Hamada, Hiroki Tsuruta, Yasuko Mori

**Affiliations:** 1 Division of Clinical Virology, Kobe University Graduate School of Medicine, Kobe, Japan; 2 Faculty of Pharmacy, Suez Canal University, Ismailia, Egypt; 3 Center for Applied Structural Science, Center for Collaborative Research and Technology Development, Kobe University, Kobe, Japan; University of California at Los Angeles, UNITED STATES

## Abstract

The tegument protein U14 of human herpesvirus 6B (HHV-6B) constitutes the viral virion structure and is essential for viral growth. To define the characteristics and functions of U14, we determined the crystal structure of the N-terminal domain of HHV-6B U14 (U14-NTD) at 1.85 Å resolution. U14-NTD forms an elongated helix-rich fold with a protruding β hairpin. U14-NTD exists as a dimer exhibiting broad electrostatic interactions and a network of hydrogen bonds. This is first report of the crystal structure and dimerization of HHV-6B U14. The surface of the U14-NTD dimer reveals multiple clusters of negatively- and positively-charged residues that coincide with potential functional sites of U14. Three successive residues, L424, E425 and V426, which relate to viral growth, reside on the β hairpin close to the dimer's two-fold axis. The hydrophobic side-chains of L424 and V426 that constitute a part of a hydrophobic patch are solvent-exposed, indicating the possibility that the β hairpin region is a key functional site of HHV-6 U14. Structure-based sequence comparison suggests that U14-NTD corresponds to the core fold conserved among U14 homologs, human herpesvirus 7 U14, and human cytomegalovirus UL25 and UL35, although dimerization appears to be a specific feature of the U14 group.

## Author Summary

Human herpesvirus 6B (HHV-6B), a causative agent of exanthema subitum for children and immunocompromised adults, encodes numerous tegument proteins that constitute the viral matrix. HHV-6B U14 is a tegument protein essential for viral propagation, and additionally it interacts with host factors such as tumor suppressor p53 and cellular protein EDD, thereby regulating host cell responses. Here, we report the molecular structure of HHV-6B U14 at an atomic resolution. The N-terminal domain of U14 (U14-NTD) adopts an elongated, helix-rich fold without any significant overall similarity to known structures. U14-NTD forms a 100 kDa homodimer through electrostatic interactions and a wide hydrogen bond network. The U14-NTD homodimer displays four clusters of electrostatic potential with deep grooves, implying multiple binding sites for other viral or host proteins. U14-NTD corresponds to the core fold shared by homologous proteins of human herpesvirus 7 (HHV-7) and of human cytomegalovirus, although dimerization seems to be specific to HHV-6 and HHV-7. The U14-NTD structure provides clues to promote further analysis on the role and behavior of U14 in the pathogenesis of HHV-6. It also leads to a comprehensive understanding of the U14 homologs in beta herpesviruses, and furthermore contributes to the overall knowledge about tegument proteins in herpesviruses.

## Introduction

Human herpesvirus 6B (HHV-6B) and the closely-related virus HHV-6A are classified as Roseolovirus genus of beta herpesvirus subfamily [1] [2] [3] [4], which also includes human herpesvirus 7 (HHV-7) and human cytomegalovirus (HCMV). HHV-6B is a causative agent of exanthema subitum for children [5] [6] by primary infection and for immunocompromised adults by reactivation of the latent virus. Diseases induced by HHV-6 primary or reactivated infection are sometimes severe, causing encephalitis [7] [8].

Herpesviruses share a common architecture of the virion that is enveloped and contains the double-stranded DNA genome in a protein shell known as capsid. The space between the envelope and the capsid is filled with a pool of tegument proteins [9] [10]. The composition of tegument proteins differs among herpesviruses, and numerous tegument proteins have been identified for HHV-6B [11]. Tegument proteins are versatile proteins suggested to have additional functions other than acting as structural components of the viral tegument [12–14]. Thus, the characteristics and function of each tegument protein remain to be defined and could have a role in understanding herpesvirus pathogenesis.

HHV-6B U14 is a tegument protein that is 604 amino acid residues in length. U14 belongs to the herpes pp85 superfamily that is shared among beta herpesviruses, and has no homologs in alpha and gamma herpesvirus [11] [15] [16] [17]. HHV-6A, HHV-6B, and HHV-7 have U14 with relatively high sequence homology. Other members of the beta herpesvirus subfamily, including HCMV, have two tegument proteins belonging to the pp85 superfamily, UL25 and UL35 [18], although their sequence identities with U14 is less than 20%. Recently, HHV-6A U14 was revealed as an essential factor in the viral life cycle because a three amino-acid deletion in the U14 sequence resulted in a defect in viral growth [19]. In addition, U14 of HHV-6A and HHV-6B associate with the tumor suppressor protein p53 in the nucleus and cytoplasm, finally being incorporated into virions with p53 [20]. Furthermore, we found that HHV-6A U14 induces cell cycle arrest in G2/M phase by associating with a cellular protein, EDD during early phase of infection [21]. These results indicate that HHV-6 U14 functions not only as a virion tegument protein, but also in viral DNA replication cycles, suggesting that it is a multi-functional protein.

Structure determination of tegument proteins is an effective approach, providing information about their structural characteristics as well as a basis for mapping the results of biochemical experiments. In this study, we solved the crystal structure of the N-terminal region of U14 protein derived from HHV-6B. The structure represents a characteristic dimer form with potential functional sites. Through sequence comparison with HHV-6A U14, HHV-7 U14, and HCMV UL25 and UL35, shared and specific features among these homologs are discussed.

## Results

### Purification and structure determination of U14-NTD

**Fig 1:**
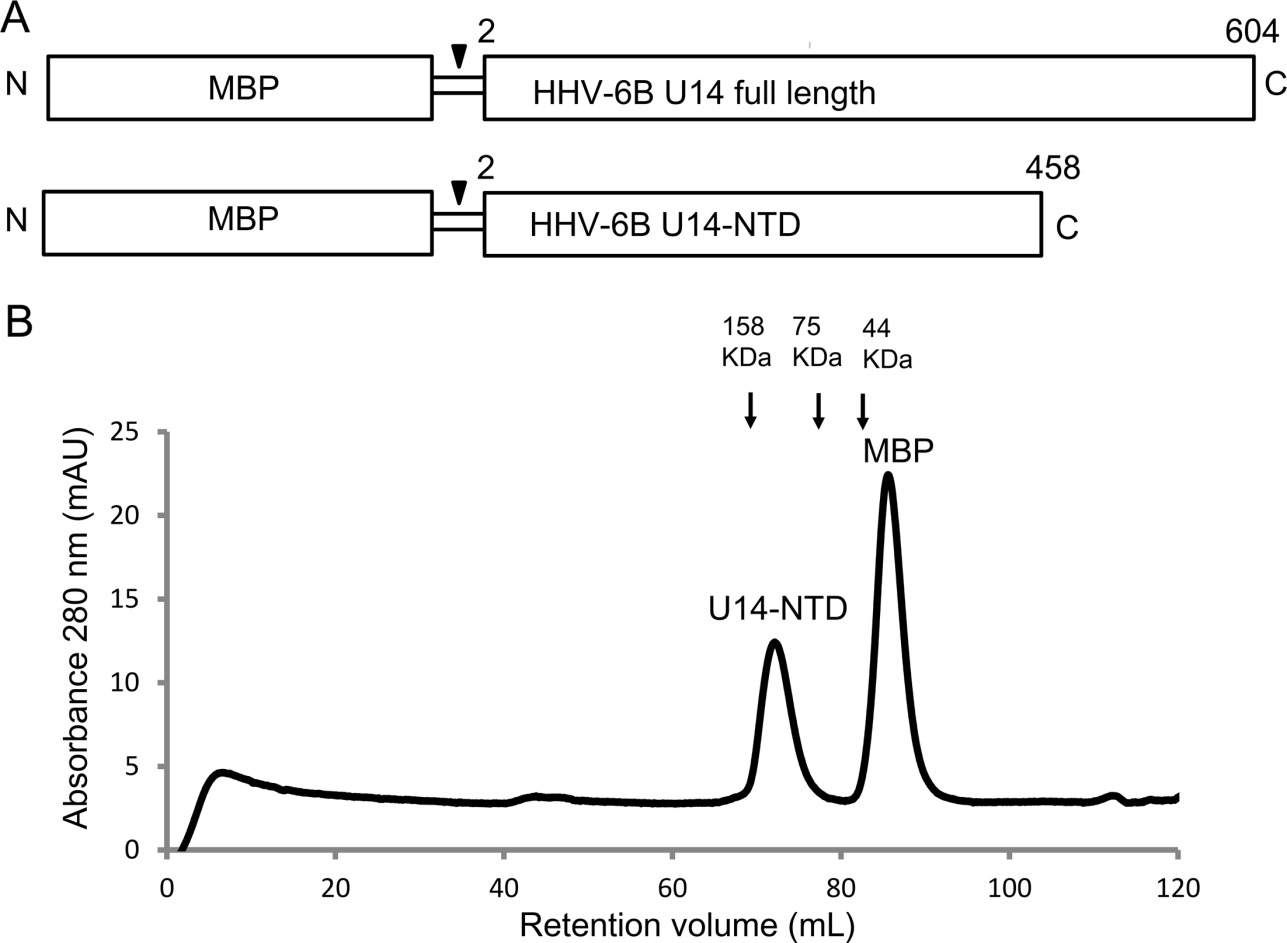
Construct of HHV-6B U14-NTD. (A) MBP-U14 and MBP-U14-NTD constructs. Arrow heads represent the HRV3c cleavage site. (B) Size-exclusion column chromatography of U14-NTD and MBP. U14-NTD elutes faster than MBP despite the similar molecular sizes of U14-NTD (50 kDa) and MBP (42 kDa). The retention volumes of standard proteins were indicated by arrows.

Full-length HHV-6B U14 (603 amino acids) was expressed in *E*. *coli* with MBP at the N-terminus (U14-MBP; Fig 1A). During purification, a fraction of U14-MBP was degraded to a smaller size, indicating that the C-terminal region of U14-MBP is unstable in *E*. *coli* (S1 Fig). Thus, a new construct was designed to express the U14-NTD corresponding to the N-terminal region (residues 2–458) in the form of an N-terminal MBP fusion (Fig 1A). MBP-U14-NTD was not degraded significantly during purification (S1 Fig), supporting the assumption that C-terminal region of MBP-U14 was degraded. Actually, the size of MBP-U14-NTD was similar to the degradation product of U14-MBP (S1 Fig). In the size-exclusion column chromatography experiment, the retention time of U14-NTD was shorter than expected from its size (50 kDa), indicating that U14-NTD forms a multimer in solution (Fig 1B). The size of U14-NTD estimated from the calibration curve was 118 kDa, which is slightly higher than the calculated size 100 kDa for a U14-NTD dimer.

Because there was no available structural information of any protein with high sequence homology to HHV-6B U14, a SeMet-derivative of U14-NTD was prepared to solve the phase problem by anomalous dispersion method. The structures of native U14-NTD and the SeMet-derivative were determined at 1.85 Å and 2.3 å resolutions, respectively (Table 1). There were two almost identical U14-NTD molecules in the asymmetric unit. Their RMS deviation for main-chain atoms and heavy atoms were 0.51 å and 1.09 å, respectively. Almost all of the U14-NTD residues were assigned to the electron density, with the exception of N-terminal residues 1–7 and C-terminal residues 456–458, indicating that the designed U14-NTD represents an actual structural domain of U14.

**Table 1:** Data collection and refinement statistics.

Parameter			U14_SeMet	U14_Native
Data Collection				
	Wavelength (Å)		0.979080	1.000000
	Space group		*P*2_1_2_1_2	*P*2_1_2_1_2
	Unit cell parameters			
		a, b, c (Å)	98.94, 211.33, 51.53	99.33, 211.53, 51.78
		α, β, γ (°)	90, 90, 90	90, 90, 90
	Resolution (Å)^a^		48.17–2.30 (2.39–2.30)	46.68–1.85 (1.92–1.85)
	Total reflections^a^		708452 (64553)	674106 (67287)
	Unique reflections^a^		48916 (4806)	93926 (9230)
	*R*_merge_ ^a^		0.158 (1.017)	0.08735 (0.575)
	*R*_meas_ ^a^		0.164	0.0945
	Mean I/sigma (I)^a^		17.93 (3.78)	18.04 (3.39)
	Completeness (%)^a^		99.95 (99.63)	99.75 (99.13)
	Multiplicity^a^		14.5 (13.4)	7.2 (7.3)
	CC_1/2_ ^a^		0.999 (0.872)	0.997 (0.872)
Refinement				
	*R*_work_ ^a^		0.1709 (0.2198)	0.1798 (0.2344)
	*R*_free_ ^a^ ^b^		0.2260 (0.2608)	0.2328 (0.2529)
	Number of atoms			
	Macromolecules		7236	7236
	Water		401	1220
	Average B-factor (Å^2^)			
		Macromolecules	37.40	23.00
		Solvent	38.00	31.80
	RMS deviations			
		Bonds (Å)	0.008	0.007
		Angles (°)	1.07	1.00
	Ramachandran plot			
		Favoured (%)	98.0	99.0
		Outliers (%)	0.22	0.11

^a^Values in parenthesis are for the outermost resolution shell.

^b^ *R*_free_ was calculated for 2,000 reflections excluded from refinement.

### Structure description

**Fig 2:**
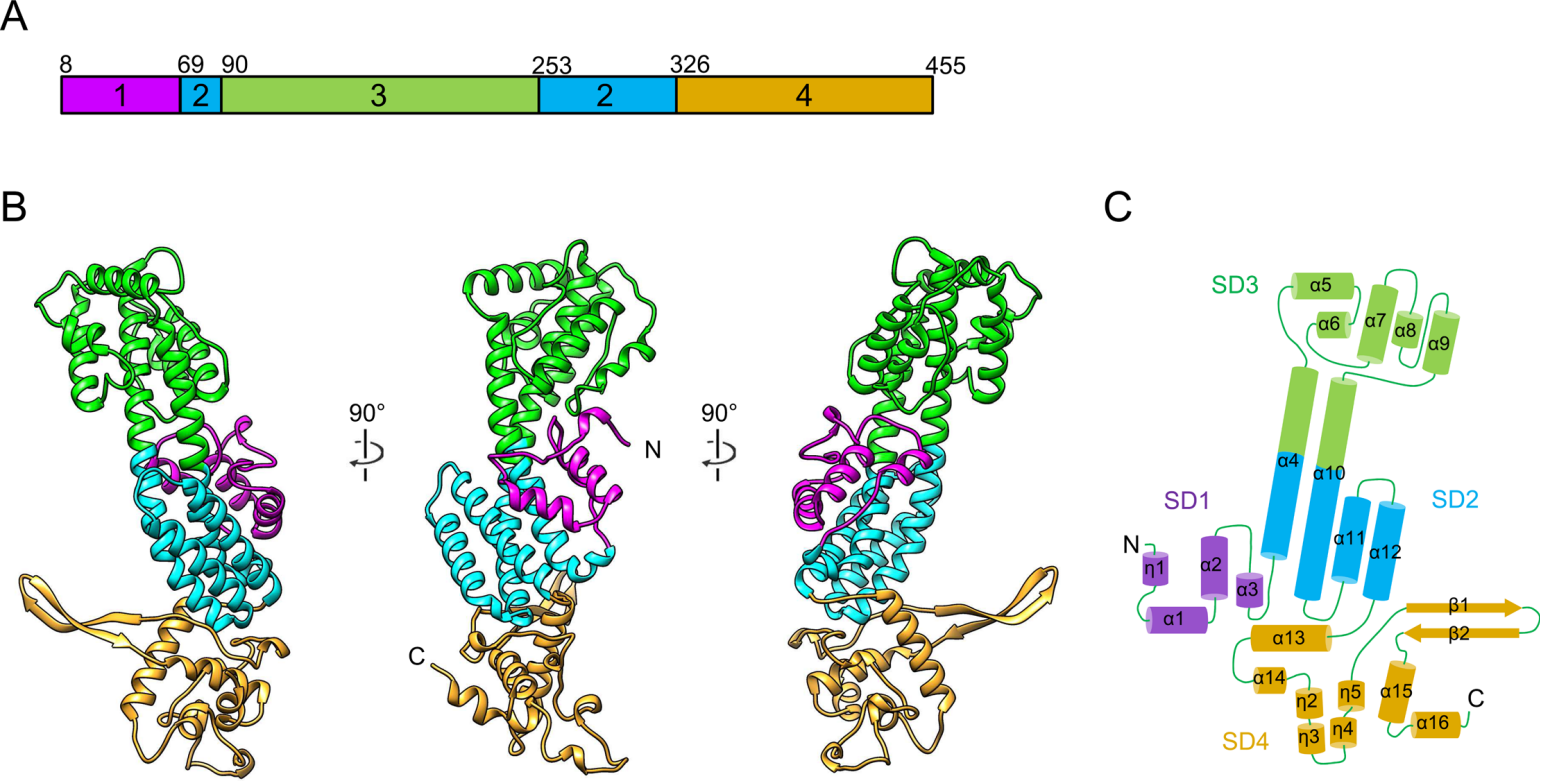
Crystal structure of U14-NTD. (A) The subdomains (SDs) of U14-NTD. Coloring is according to SDs as follows, SD1: magenta, SD2: cyan, SD3: green, and SD4: khaki. (B) Ribbon representation of the U14-NTD monomer. (C) Secondary structure diagram of U14-NTD. Cylinders and arrows represent α/3_10_ helices and β strands, respectively.

U14-NTD has an elongated helix-rich structure composed of sixteen α helices, four 3_10_ helices and two β strands. To facilitate structure description, the U14-NTD structure was divided into four subdomains (SDs) based on secondary structure topology and spatial arrangement (Fig 2). The four-helix bundle SD2, which is composed of the N-terminal half of α4, the C-terminal half of α10, α11 and α12, forms a central part of U14-NTD (Fig 2, cyan). At the preceding N-terminal region, SD1 forms a compact fold including helices η1, α1, α2 and α3 (Fig 2, magenta), and is associated with SD2. SD3 is located at one side of the elongated long axis of U14-NTD (Fig 2, green). The C-terminal half of α4 and the N-terminal half of α10 are surrounded by five α helices (α5, α6, α7, α8, and α9) and form a compact fold of SD3. At the opposite side of the long axis, the C-terminal region of U14-NTD folds as SD4 composed of α13, α14, η2, η3, η4, η5, β1, β2, α15 and α16 (Fig 2, yellow). The β1 and β2 form a recognizable β hairpin that protrudes from the core overall fold.

Analysis by the DALI program [22] with the latest set of Protein Data Bank (PDB, www.rcsb.org, [23]) entries revealed that SD2 is similar to a variety of proteins characterized by four-helix bundles (Table 2). In addition, SD3 showed marginal similarity to Unc-51-like kinase 3 and other proteins (Table 2). For SD1 and SD4, no significant homology to known proteins was detected.

**Table 2:** Structural similarity for U14-NTD subdomains suggested by Dali analysis.

U14-NTD subdomain	Registered protein name	Z-score	RMSD(Å)	No. of aligned residues	% identity	PDB ID
SD2	Regulator of Sigma D	7.6	3.6	121	7	2p7v
	Programmed Cell Death Protein	7.5	3.3	117	9	3l8i
	Hemerythrin	6.2	3.2	102	8	1hmd
	Vinculin	6.2	3.9	129	13	1rke
	Protein Tyrosine kinase 2 beta	6.2	3.8	115	6	3gm1
	Focal Adhesion kinase 1	5.9	3.5	105	7	1ow8
	Bacteriorhodopsin	5.8	3.8	123	7	2brd
	Alpha E-Catenin	5.6	4.4	113	9	1l7c
	Copper Efflux ATPase	5.4	3.6	90	4	4bbj
	CRK associated substrate	5.3	4.1	114	8	1z23
	Vacuolar protein sorting-associated protein 28	5.1	2.6	84	11	2j9u
	Signal Transduction Histidine Protein kinase	5.1	2.6	85	15	3iqt
SD3	Unc-51-like kinase 3	4.8	2.8	87	3	4wzx
	Uncharacterized protein	4.4	2.8	77	4	2rld
	Glutamate carboxypeptidase II	4.3	3.8	96	6	2c6g
	Retinoic acid inducibleprotein I	4.2	5.1	71	10	4a2w
	Endoribonuclease dicer	4.0	2.7	71	8	4wyg

### U14 NTD forms a homodimer by broad interactions

**Fig 3:**
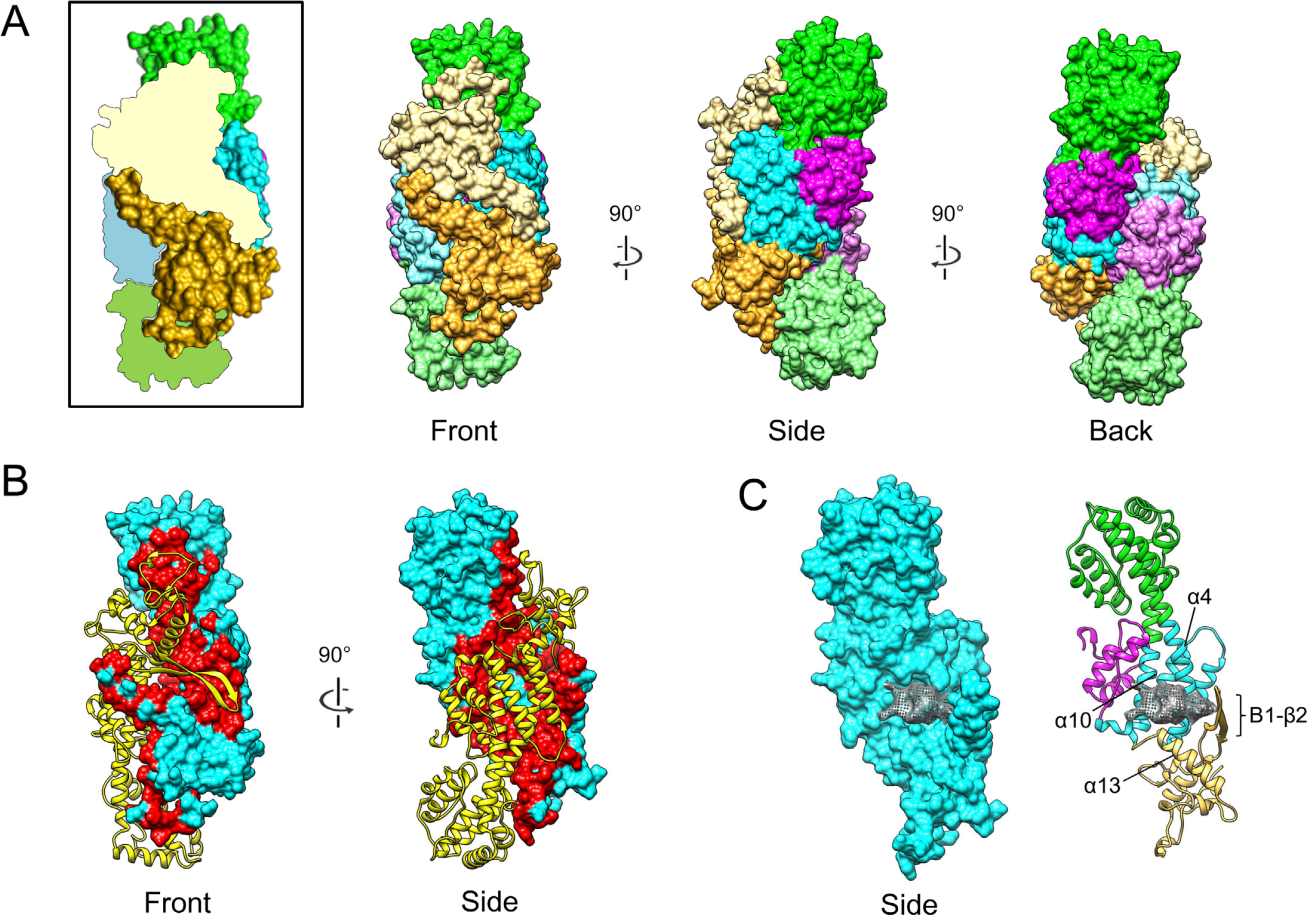
The dimeric form of U14-NTD. (A) Surface representation of the U14-NTD dimer. For reference, a monomer with a schematic partner is shown at the left in the box. The surface is colored according to the subdomains (SDs), SD1: magenta, SD2: cyan, SD3: green, and SD4: khaki. To distinguish the two molecules, light colors were used for one monomer. (B) Dimerization interface. For simplicity, one of the monomers is shown as a surface model colored in cyan and its contact region is colored in red. The other monomer is represented as a ribbon model colored in yellow. (C) The cavity within the dimer interface is shown as a gray mesh on the surface model (left) and ribbon model (right). For clarity, only one monomer is shown.

In the crystal structure, a dimer is formed along the long axis of U14-NTD in an antiparallel orientation (Fig 3). The dimer can be regarded as two right hands shaking one another with the protruding β hairpins forming the “thumbs”. A two-fold axis is located by the side of the β hairpin, resulting in an arrangement of crossed hairpins. All of the SDs are involved in the dimer interface. The calculated buried surface area per monomer is 4146 Å^2^ (Fig 3B, red), which is a relatively large value compared with those of known homodimer structures of similar size, at approximately 2800 Å^2^ [24].

One noticeable characteristic of the U14-NTD dimer is an internal cavity within the dimer interface (Fig 3C). The two-fold axis of the dimer penetrates the cavity. The volume of the cavity is approximately 1200 Å^3^ and corresponds to 2.1% of the volume of the monomer (57400 Å^3^). The internal cavity is enclosed with α4 and α10 of SD2 and α13 of SD4 from each monomer. The β hairpins of SD4s also face this cavity, forming a lid that separates it from the outer solvent.

**Fig 4:**
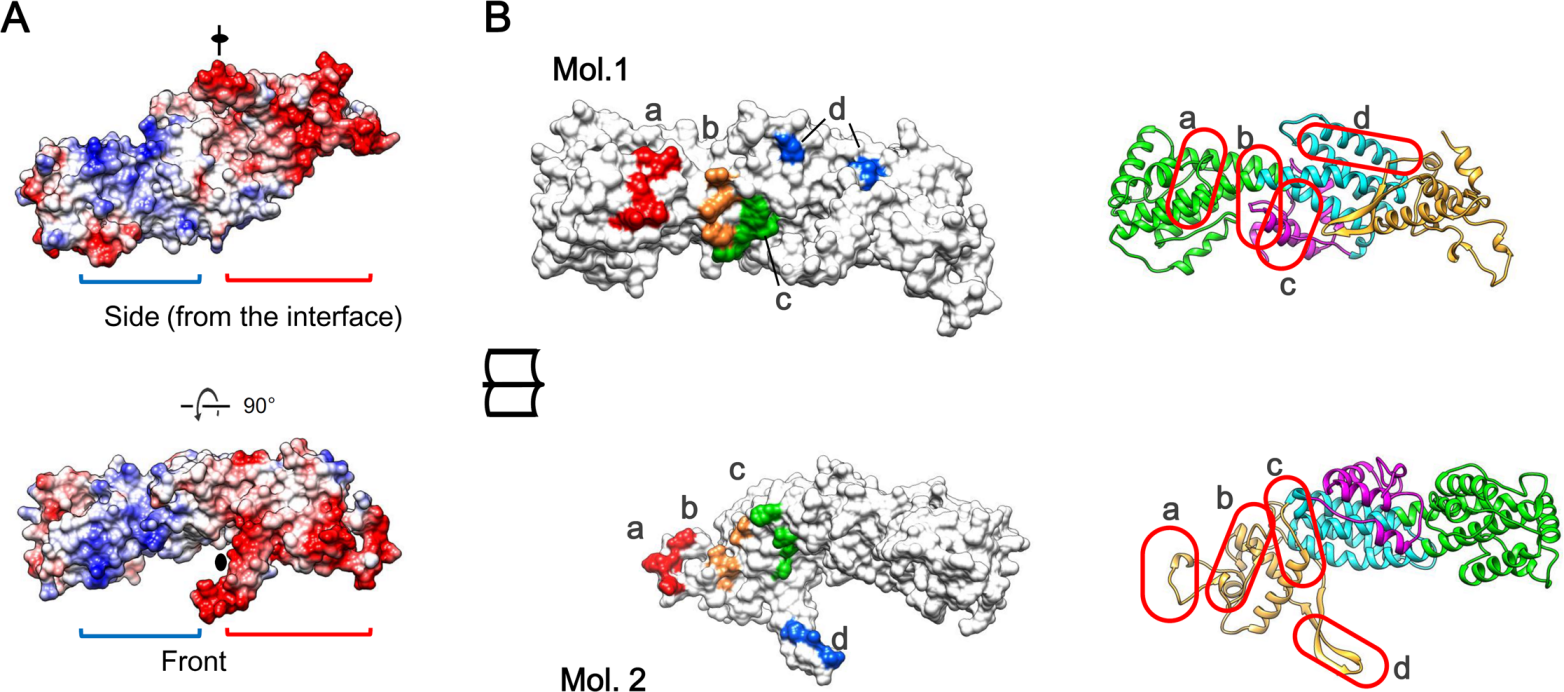
Interactions stabilizing the U14-NTD dimer. (A) Electrostatic potential of the monomer surface. The positively- and negatively-charged areas are indicated by blue and red brackets, respectively. Note that these areas are separated by the two-fold axis indicated by the black symbol. (B) Hydrogen bond clusters at the dimer interface of U14-NTD. An open-book representation of the surface models of the two monomers, named Mol.1 and Mol.2, is shown, associated with their ribbon representations on the right. The surfaces involving the four hydrogen bonds clusters a, b, c, and d are colored in red, orange, green, and blue, respectively. Their locations are also indicated in the ribbon models with red circles. Detailed interaction modes are depicted in S2 Fig and summarized in S1 Table.

Broadly spanning electrostatic interactions contribute to the dimerization (Fig 4A). At the dimer interface, a monomeric U14-NTD shows a negatively-charged surface between SD2 and SD4. On the other hand, a positively-charged surface is found between SD2 and SD3 in the same monomer. In the dimer form, the negatively-charged area of each monomer faces the positively-charged area of the opposite monomer. There are a lot of hydrogen bonds within and around the electrostatically attracting areas. Four clusters were found and named as interaction sites a, b, c, and d (Fig 4B). A total of 40 hydrogen bonds were formed in these areas, indicating tight and specific dimerization. Their distribution is summarized in S1 Table and the detailed interaction modes are shown in S2 Fig.

### Surface features of the U14-NTD dimer

**Fig 5:**
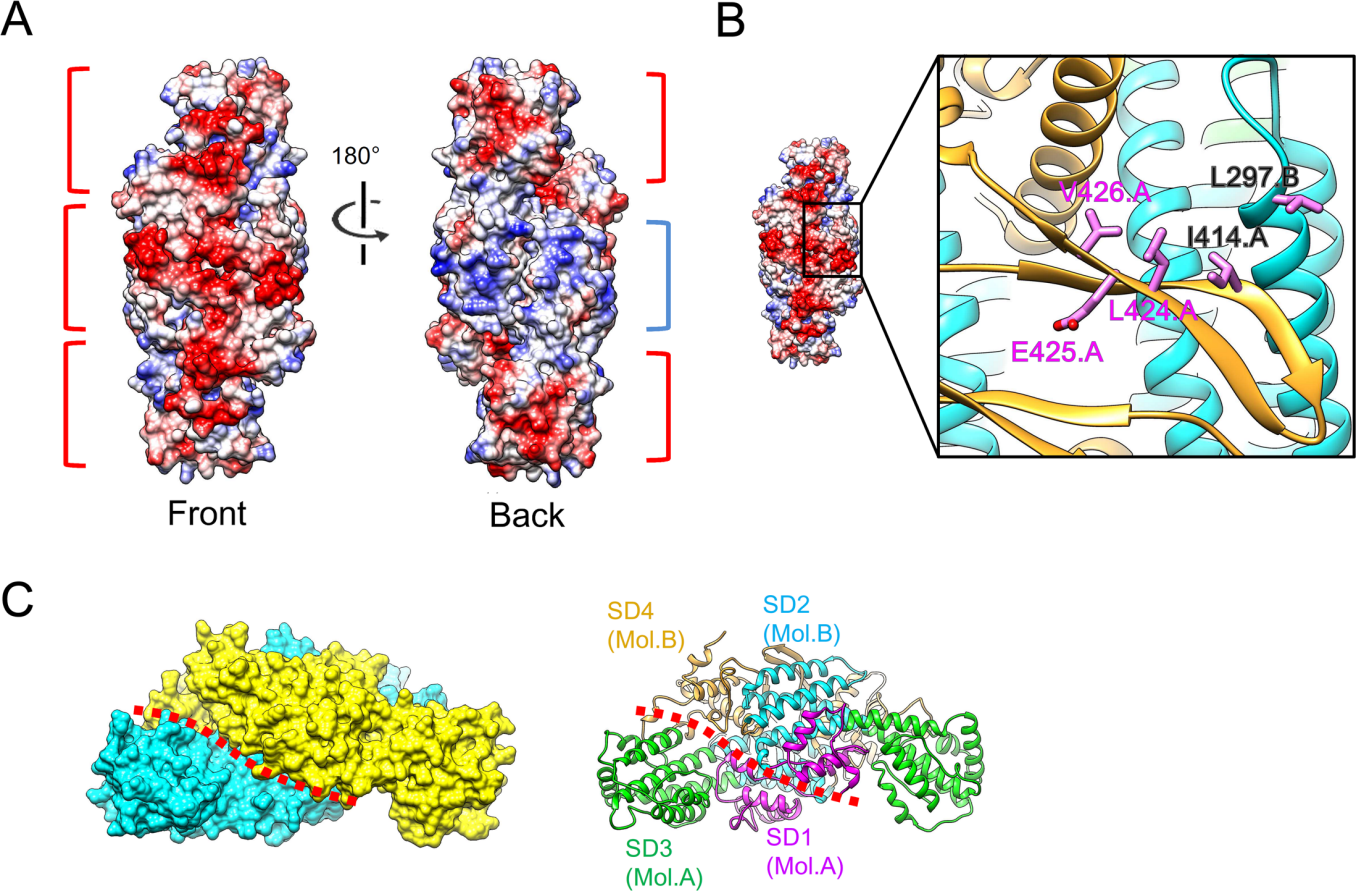
Potential functional sites on the dimer surface of U14-NTD. (A) Electrostatic potential of the U14-NTD dimer. The negatively- and positively-charged clusters are indicated by red and blue brackets, respectively. (B) The position of three amino acids, L424, E425, and V426, are shown as stick models. The adjacent residues I414 and L297 that form the hydrophobic patch are also shown. (C) Deep groove along the interface. The position of the groove is indicated by a red dotted line. For reference, a ribbon model in the same orientation is shown at the right with the positions of SD2, SD3, and SD4 indicated.

The U14-NTD dimer shows characteristic multiple clusters of positive and negative electrostatic potential on the surface (Fig 5A). At the β hairpin side, an extended negatively-charged area is formed across the two-fold symmetry axis (front side, Fig 5A, left). The dimer surface of this side is composed primarily of SD4. The β hairpin of each monomer contains six negatively-charged residues (S3A Fig). The region 342–378 of SD4, which corresponds to the outermost part of the long axis of U14-NTD, includes 11 negatively-charged residues (S3B Fig). On the opposite side (back side, Fig 5A right), the area around the two-fold axis is surrounded by seven positively-charged residues from each monomer (S3D Fig). At this same back side, a negatively-charged cluster consisting of ten negatively-charged residues is observed at the peripheral area distant from the two-fold axis (S3C Fig).

The three amino acids, L424, E425, and V426, of which deletion or substitutions to alanines caused a defect in viral growth [19], were mapped to the β hairpin (Fig 5B). The side-chains of L424 and V426 face the solvent side and constitute a continuous hydrophobic patch with the side-chain of I414 on the opposite β strand and the side-chain of L297 on SD2 of the partner monomer (Fig 5B).

In contrast to the flat surface of the front side, the back side has deep grooves along the dimer interface due to the staggered arrangement of monomers (Fig 5C). One side of the groove is exclusively composed of SD3, with SD4 and SD2 of the partner monomer forming the opposite wall. The length, depth, and width (distance between monomers) were roughly estimated to be ~30 Å, ~20 Å, and ~20 Å, respectively (S4 Fig).

### HHV-6B U14-NTD is conserved between U14 and its homologs

**Fig 6:**
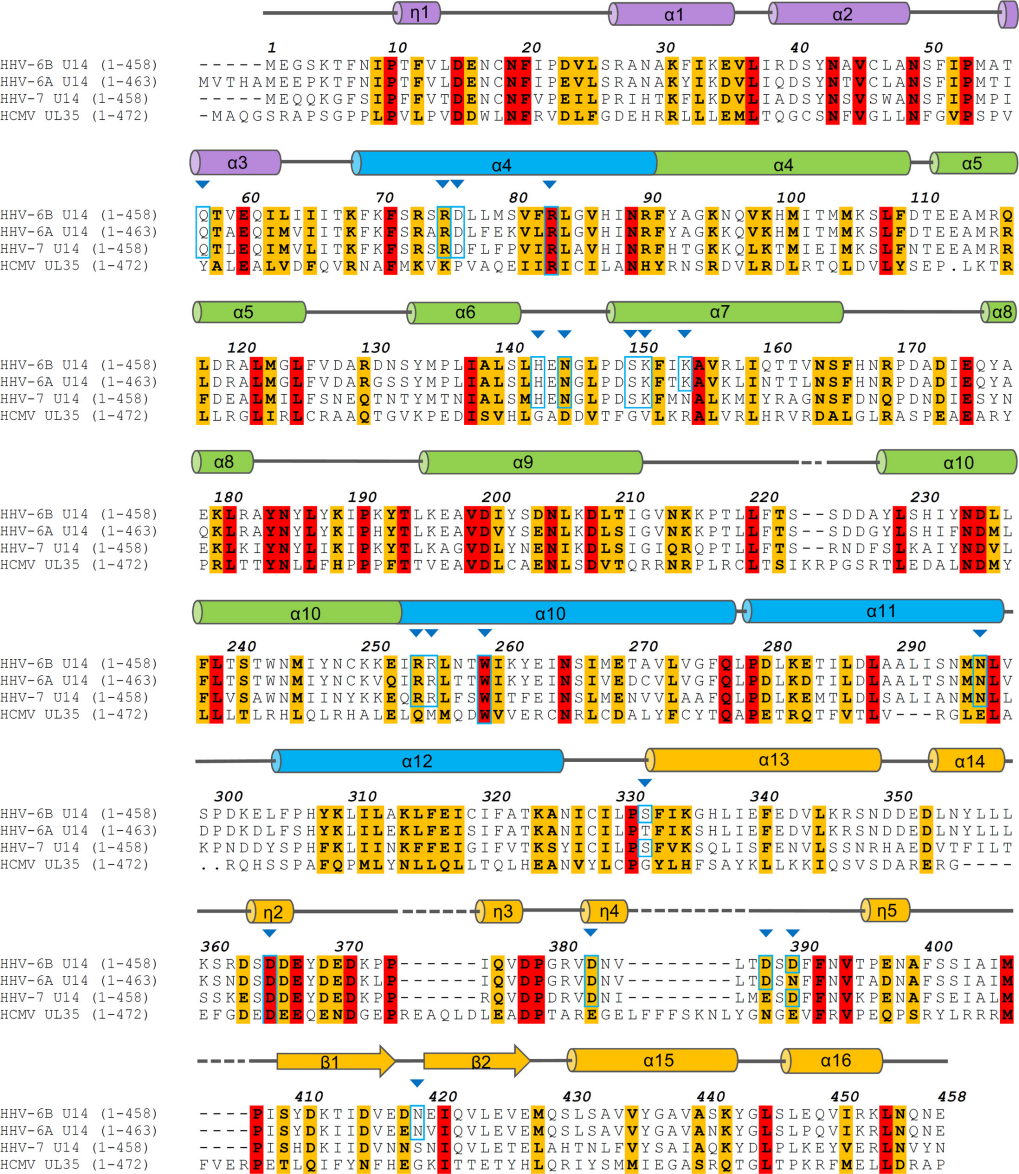
Multiple sequence alignment of HHV-6B U14 and its homologs combined with the structural information of U14-NTD. The sequences of HHV-6A U14, HHV-7 U14, and HCMV UL35 are aligned. For clarity, only the region corresponding to U14-NTD is shown. The cylinders and arrows above the alignment represent α/3_10_ helices and β strands of U14-NTD, respectively. Identical and similar residues among all sequences are highlighted in red and orange, respectively. The blue arrowheads indicate the positions of residues whose side-chains participate in the U14-NTD dimer hydrogen bond network. At these positions, the cyan boxes indicate the residues identical to those of HHV-6B U14. Clustal Omega [25] was used for the alignment. Accession numbers for the sequences are as follows: HHV-6B U14: gi|4996002, HHV-6A U14:gi|9628315, HHV-7 U14: gi|1139615, and HCMV UL35: gi|270355798.

To address issues of similarity and difference between HHV-6B U14 and its homologs, multiple sequence alignment was performed for HHV-6B U14, HHV-6A U14, HHV-7 U14, and HCMV UL35 (Fig 6). The alignment combined with the structural information of U14-NTD showed that U14-NTD is a core part conserved among all members. In the core region, HHV-6A U14 and HHV-7 U14 are well aligned with HHV-6B U14 across all SDs. HCMV UL35 was also aligned in the core part, except for the SD4, where short gaps are required for the alignment (Fig 6). For HCMV UL25, another pp85 family protein, similar alignment was obtained, although the pattern is different from that of HCMV UL35 (S5 Fig). These alignments suggest that these U14 homologs have a similar helix-rich fold at the core region.

Next, we examined the conservation of the residues involved in the hydrogen bond network in the HHV-6B U14-NTD dimer. Most of the HHV-6B U14-NTD dimer interaction sites are occupied by identical residues in HHV-6A U14 and HHV-7 U14, indicating that these homologs also dimerize in a similar manner. Of the 19 residues whose side-chains involved in the interaction, 17 and 16 residues are identical for HHV-6A U14 and HHV-7 U14, respectively (Fig 6). By contrast, the residues participating in the dimer interface are different from HHV-6B U14 in HCMV UL25 and UL35. For HCMV UL25 and UL35, only 4 and 3 residues are identical to HHV-6B, respectively. It suggests that HCMV UL25 and UL35 takes a different form to that of the HHV-6B U14-NTD dimer.

## Discussion

The HHV-6B U14-NTD structure, comprised of residues 2–458, reveals a helix-rich fold forming a compact homodimer. The broad and intricate interactions between each monomer (Figs 3 and 4), as well as the retention time of the size-exclusion column chromatography (Fig 1B), support the suggestion that a dimer is the natural form for U14-NTD. Multimerization of viral proteins has been frequently reported, particularly for structural proteins constituting the capsid and associated proteins. A number of tegument proteins have also been shown to form self-associated multimers, such as HSV-1 UL36 [26] and VP22 [27], HCMV pp65 [28] and pp28 [29], and murine gammaherpesvirus 68 ORF52 [30]. Compared with these, the ~50 kDa U14-NTD is relatively large as a dimerization domain with a broad interface in which all four SDs are included. Although the viral matrix is considered to be an amorphous/disordered protein pool in general, multimerization of its constituents would impose local order to some extent as a corollary to the symmetries of their own and of their interaction sites for other partners. Such local order in the viral matrix has been suggested for matrix proteins of RNA virus; multimerization of matrix proteins relates to the formation of a protein lattice in the matrix and contributes to the membrane deformation required for the budding process [31], [32]. Thus, the dimerization of U14 revealed in this research implies a role for this protein as a scaffold in the viral matrix. Analyzing the expression amount of U14 protein in virions would be required. As far as we know, the expression amount of HHV-6 U14 has not been investigated, hence it should be addressed in a future research. In the case of HHV-7, U14 is known as a major antigen pp85 [33], and U14 is thought to be relatively expressed abundantly. On the other hand, one of predominant major antigens of HHV-6 has been shown to be U11 [34], [35], which has been revealed to interact with U14 [36]. It may be noteworthy to mention that the HCMV UL25 was expressed abundantly especially in the dense body [37].

The HHV-6B U14 structure suggests multiple structural features as potential function sites, such as the negatively- and positively-charged clusters (Fig 5A), the β hairpin flanked by the hydrophobic patch (Fig 5B), and the grooves formed along the dimer interface (Fig 5C). These distinct structural features would be consistent with the multiple functions of U14. U14 is observed in at least three different locations during the protein's life cycle, namely in the nucleus of a host cell at an early phase of infection, in the cytoplasm at a late phase, and in the virion [20]. At each location, U14 could have a different role via interaction with different host/viral factors. Thus far, at least two associated host proteins are reported for U14: the tumor suppressor p53 [20] and EDD [21]. Experiments using deletion mutants of U14 revealed that three amino acids on the β hairpin and the C-terminal region outside U14-NTD are implicated in the interaction with p53 and EDD, respectively [21]. Among the potential function sites, the area around the β hairpin is of importance because substitution or deletion of three amino acids (L424, E425, and V426) on the β hairpin results in a defect in viral multiplication [19]. It is expected that the deletion of the three amino acids strongly affects the β hairpin structure due to the imbalanced length of the two β strands. The β hairpin contributes to the dimer interaction (Fig 4 and S2D Fig); such deletion could change either the fold of U14-NTD or its dimerization and function. On the other hand, substitutions probably maintain the β hairpin structure because the original side chains are not involved in folding and easily simulated to be substituted without any necessity to change its structure. To further assess the importance of the β hairpin, we performed immunoprecipitation assay with HHV-6A U14 mutants in which residues on the β hairpin were substituted (S6 Fig). The p53 interaction was abolished by the deletion of three amino acids corresponding to L424, E425 and V426 as reported previously [21]. In contrast, a single alanine substitution at the corresponding position to I414 (Fig 5B) did not affect the interaction. Therefore, p53 is suggested to be sensitive to the change in β hairpin structure due to the deletion. Another possibility is that the β hairpin is involved in binding to other viral proteins, such as tegument proteins to form the tegument structure or capsid or envelope proteins to form the virion structure. Recently, we identified a major tegument U11 as the binding partner of U14 [36], then the effect of the mutation/deletion on the β hairpin was also analyzed by the immunoprecipitation assay (S6 Fig). The interaction between U14 and U11 was abolished by the deletion of the corresponding residues of the L424, E425 and V426. Moreover, in contrast to p53, a single substitution at the corresponding residue of I414 (I414A) caused impaired interaction with U11. Thus, we suggest that the β hairpin is the binding site for U11 and the exposed hydrophobic sidechain of I414 observed in the U14-NTD structure is likely to be recognized by U11. Because U11 is an abundant and essential tegument protein of HHV-6 [36], further research focused on the interaction via the β hairpin will provide more information about the functionality of U14.

**Fig 7:**
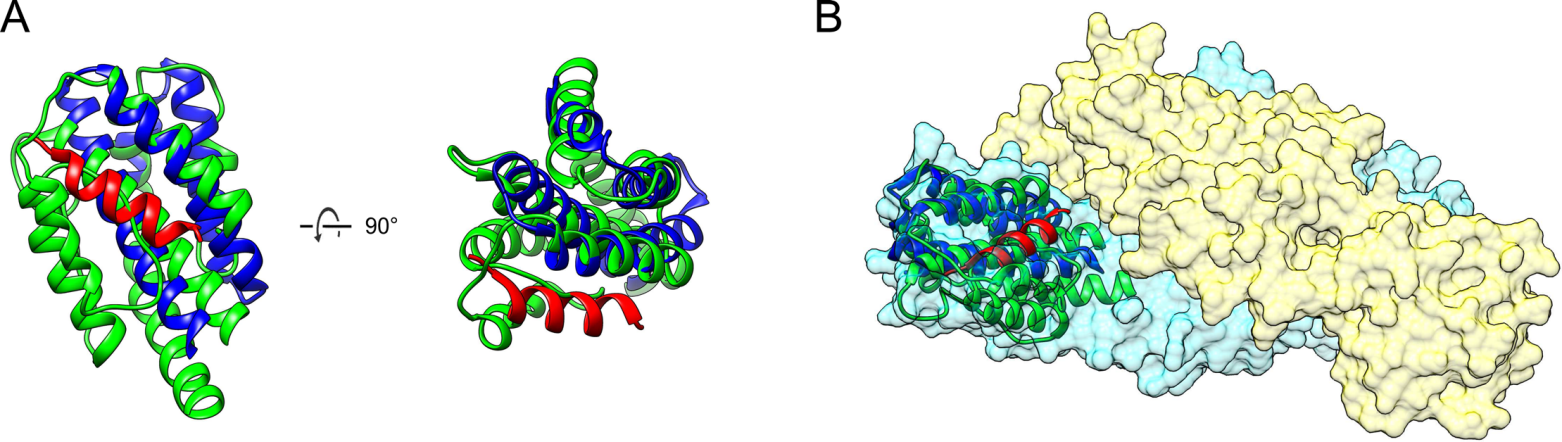
Structural similarity between the Unc-51-like kinase 3 (ULK3) MIT domain and U14-NTD SD3. (A) The coordinates of the ULK3 MIT domain (blue) and MIM peptide (red) are superposed on U14-NTD SD3 (green). (B) The MIM binding site of ULK3 MIT corresponds to the U14-NTD SD3 surface that is located at the deep cleft between two monomers.

SD3 of U14-NTD showed structural similarity with the MIT (microtubule interacting and trafficking) domain of Unc-51-like kinase 3 (ULK3, PDB ID: 4wzx, [38]) by DALI analysis (Table 2 and Fig 7A). Recent research revealed that the ULK3-MIT domain interacts with the MIM2 motif of ESCRT-III and phosphorylates the site, resulting in inhibition of cytokinesis during the cell division process [38]. The MIM2 binding site of the ULK3-MIT domain was superposed to a part in the deep groove observed around U14-NTD SD3, and partially opened to the solvent (Fig 7B). Thus, it is tempting to speculate that SD3 and the nearby groove of U14-NTD dimer serve as the binding site for ESCRT-III or related proteins, thereby contributing to their transporting function. Considering that ESCRT-III is involved in the viral maturation/budding step [39] [40], further experiments are required to examine the relationship between U14 and the ESCRT system, thus further elucidating U14 function.

Structure-based sequence analysis revealed that the HHV-6B U14-NTD corresponds to the core part conserved between U14 and UL25/UL35 (Fig 6 and S5 Fig). The similarity across this region indicates that HHV-6A U14, HHV-7 U14, and HCMV UL25 and UL35 adopt the same elongated helix-rich fold. However, the absence of homology in HCMV UL25 and UL35 at the dimer interface region of HHV-6B U14 suggests that the dimer form is specific to U14 proteins. Because most of the structural features that likely constitute the functional sites of U14 depend on the dimer form, this information is not applicable to HCMV UL25 and UL35. The C-terminal region outside U14-NTD contains a large proportion of hydrophilic and glycine residues (S2 Table). This indicates that U14 consists of the core part with an intrinsically disordered tail [41]. The C-terminal region following the core fold differs in length among U14 and U25/U35 proteins (HHV-6B U14: 147 residues, HHV-6A U14: 146 residues, HHV-7 U14: 190 residues, HCMV UL25: 13 residues, and HCMV U35: 169 residues), posing difficulty in obtaining valid sequence alignments. The variation in the C-terminal region has been indicated from the alignment between HHV-6A and HHV-7 U14 [33]. HCMV UL25 has a long extension of 180 residues that precede the core part instead of the C-terminal region observed in other homologs. The amino acid compositions of these extensions share a common propensity. Similar to the HHV-6B U14 C-terminal region, those of other homologs are dominantly composed of hydrophilic and glycine residues (S2 Table). This indicates that these regions are intrinsically disordered without a stable structure in solution [41], and thus the construction, a core fold followed by an unstructured tail(s), is common for these proteins of the pp85 family. Interestingly, the proportion of serine residues is unusually high, around 20%, in these tail regions (S2 Table), which suggests that the tail could be the site of post-translational modifications such as phosphorylation and glycosylation. The importance of the C-terminal region has been established for U14 and UL35. The C-terminal region of HHV-6 U14 is required for interaction with EDD and, subsequently, cell cycle arrest [21]. HCMV UL35 has an isoform, UL35A, corresponding to the C-terminal 193 amino acids of UL35. UL35A functions to modulate expression of immediate early genes [18]. Alignment between HHV-6B U14 and HCMV UL35 showed that UL35A includes only a part of α15 and α16 in SD4, suggesting that UL35A is unlikely to form a stable fold on its own. The structural information derived from U14-NTD provides the basis for further structure-based analyses necessary for addressing the roles of these similar, but significantly different, tegument proteins.

## Materials and Methods

### Cloning

The coding sequences for the U14 N-Terminal Domain (U14-NTD) was amplified by PCR from optimized viral DNA (optimized by GeneOptimizer) of HHV-6B strain HST using the U14 forward primer, with the HRV 3C protease site underlined, (5’- ACAGGATCCCTGGAGGTGCTGTTCCAGGGCCCCGAAGGCAGCAAGACCTTC-3’) and the U14 reverse primer (3’-ACAGTCGACTTACTCGTTCTGGTTCAGC-5’). The PCR product was subcloned into pMAL-C2 using BamHI and SalI restriction sites. The cloned DNA fragment was sequenced with a 3130 Genetic Analyzer (Applied Biosystems).

### Expression of U14

For native U14-NTD, freshly transformed *Escherichia coli* strain BL21 cells were incubated at 37°C overnight in 10 ml lysogeny broth (LB) starter culture supplemented with 50 μg ml^-1^ carbenicillin. The starter culture was diluted into 1 liter LB medium supplemented with 50 μg ml^-1^ carbenicillin and grown at 37°C until an OD_600_ of 0.6–0.7. Then the temperature was shifted to 20°C and the cells were induced with 0.3 mM isopropyl-β-_D_-thiogalactopyranoside (IPTG). The expression was induced for 24 h.

To prepare a selenomethionine (SeMet) derivative of U14-NTD for phase determination, *Escherichia coli* strain B_834_ was used as a host. Cells grown overnight in 10 ml LB medium were then diluted into 400 ml LB medium supplemented with 50 μg ml^-1^ carbenicillin and grown at 37°C until an OD_600_ of 0.9–1.0. Cells were harvested by centrifugation and suspended in SeMet M9 medium supplemented with 50 μg ml^-1^ carbenicillin. The final volume of medium was 1 liter when the main culture was started. Cells were grown at 37°C until the OD_600_ reached 0.6–0.7 before being induced with 0.3 mM IPTG. The expression was induced for 16–20 h.

### Purification

Cells containing native U14-NTD or SeMet U14-NTD were harvested by centrifugation at 8000 ×*g* for 12 min at 4°C and suspended in column buffer (20 mM TrisHCl pH 7.4, 200 mM NaCl, and 0.1 mM DTT) with 0.5% v/v TritonX-100. The lysate was stored at -80°C for 12–14 h, and then disrupted by sonication. Insoluble proteins were removed by centrifugation at 11000 ×*g* for 15 min at 4°C. As the pMAL-C2-encoded U14-NTD contained an N-terminal maltose-binding protein (MBP) tag, Amylose Resin (NEW ENGLAND BioLabs) was added to the supernatant and gently rocked at 4°C for 10–12 h. The Amylose Resin was spun down by centrifugation at 500 *×g* for 5 min at 4°C and washed with cold column buffer five times before being applied to a 20 ml column (BioRad). The column was washed with five column volumes of column buffer. The U14-NTD was eluted with column buffer containing 10 mM maltose. The MBP tag was removed by adding PreScission Protease (GE Healthcare; 15 U mg^-1^ U14-NTD with 0.4 mM DTT) using the HRV 3C protease site as described above. Further purification was carried out by size-exclusion chromatography using a Superdex 200pg column (GE Healthcare). The column was calibrated by HWM Calibration Kit (GE Healthcare). The protein was concentrated to 2.0–2.5 mg ml^-1^ using an Amicon Centrifugal Filter (molecular weight cut-off 30 KDa, Millipore) and the purity was assessed by SDS-polyacrylamide gel electrophoresis and Western blot using an antibody against MBP.

### Crystallization

Purified U14-NTD was passed through a 0.22 μm Ultrafree Centrifugal Filter (Millipore) to remove aggregate. The concentration of the protein was estimated based on an *A*_*280*_ of 0.75 for 1 mg ml^-1^, calculated from the amino acid composition. Initial crystallization screening of U14-NTD was executed in 96-well plates at 4°C by the sitting-drop vapor-diffusion technique using the screening kit Index HT^TM^ (Hampton Research). Each drop was prepared by mixing 0.5 μl of protein solution (both 2.5 mg and 1.25 mg ml^-1^ U14-NTD, 20 mM TrisHCl pH 8.0, 100 mM NaCl, and 0.1 mM DTT) with 0.5 μl reservoir solution, and was then equilibrated against 60 μl reservoir solution. Crystallization conditions were optimized by varying the pH, salt and precipitant concentration in 24-well plates. Finally, crystals suitable for X-ray analysis were obtained from drops prepared by mixing 1.0 μl protein solution (1.25 mg ml^-1^) with 1.0 μl reservoir solution consisting of 0.2 M Potassium sodium tartrate tetrahydrate and 16–18% w/v Polyethylene glycol 3,350 at 4°C. The crystals described here formed in 3–5 days and were harvested 30–40 days later to reach a size suitable for data collection.

### Data collection, processing, and structure determination

X-ray diffraction data were collected on beamline BL26B1 and BL26B2 at SPring-8, Harima, Japan [42]. For data collection, crystals were transferred into a solution consisting of the reservoir solution supplemented with 25% glycerol prior to being flash frozen in liquid nitrogen. The data were processed using XDS [43] and indexed in space group *P*2_1_2_1_2. Dataset of SeMet-U14-NTD was collected at the peak wavelength of Se K-edge and used for the experimental phasing by Phenix.autoSol [44,45]. Dataset of native U14-NTD was solved by molecular replacement method with Phenix.phaser [46]. The SeMet-U14-NTD model was used as the search model. Structural refinement was performed with Phenix.refine [44] [47] and Coot [48]. Structural analysis and image depiction were performed using MolMol [49] and UCSF Chimera [50]. The synchrotron radiation experiments were performed at BL26b1 and BL26b2 in SPring-8 with the approval of RIKEN (Proposal No. 2014B1234, 2015A1070, and 2015A1101). The coordinates and structure factors for the U14-NTD structure has been deposited in the Protein Data Bank under the accession number 5B1Q.

## Supporting Information

S1 Table Summary of the hydrogen bonds at the dimer interface. (DOCX)

S2 Table Propensity in the amino acid composition of U14 and homolog proteins. (DOCX)

S1 Fig U14-MBP is unstable in *E*. *coli*. U14-MBP expressed in *E*. *coli* was degraded into multiple bands. In contrast, U14-NTD-MBP was not degraded, and the size was similar to the degraded product of U14-MBP. MBP was detected by Western blotting technique with anti-MBP antibody.
(TIF)

S2 Fig Detailed views of the hydrogen bond network observed in the U14-NTD dimer. Monomers are indicated as Mol.1 (cyan) and Mol.2 (yellow). (A) Site a. The η3 and the following loop in SD4, which is located at the outermost region of the long axis, face the N-terminal region of α5 and the preceding loop in SD3 of Mol.2. At the loop region in SD4, the main-chain forms hydrogen bonds with the side-chains of N145, H143, and K151 in SD3 of Mol.2. The beta carboxyl group of D365 on the η3 forms hydrogen bonds with the side-chains of S150 and K154 of Mol.2. (B) Site b. The loop region 376–390 including η4 faces α10 and α3 of the partner molecule. The carbonyl oxygen of Q376, D378, and R381 forms hydrogen bonds with R254 of Mol.2. Three aligned aspartic acids, D383, D388, and D390 protrude from the interface and form hydrogen bonds. The side-chains of D383 and D388 form hydrogen bonds with the side-chain of R255 of Mol.2. D390 forms a hydrogen bond with Q57 in SD1 Mol.2. (C) Site c. The N-terminal tip of α4 (residues 69–79) loops back 90° and is involved in interactions with site c near the two-fold axis of the dimer. The side-chain of R75 forms hydrogen bonds with the carbonyl oxygens of E60 and Q61 in the partner molecule. D76 forms hydrogen bonds with R83 of Mol.2. This hydrogen bond is the one closest to the two-fold axis of the dimer. In the vicinity, the S332 hydroxyl group forms a hydrogen bond with the indole nitrogen of W259 in Mol.2. (D) Site d is also close to the two-fold axis, but at the opposite side of site c, around the β hairpin. At the tip of the β hairpin, the main-chain atoms of N419 and I421 form hydrogen bonds with the adjacent main-chain atoms of I405 of Mol.2. The Nδ2 group of N419 forms a hydrogen bond with the carbonyl oxygen of I403 of Mol.2. Additionally, the main-chain carbonyl oxygen of T413 is hydrogen bonded with the side-chain of N296 at α10 of Mol.2.
(TIF)

S3 Fig Detailed views of the electrostatic clusters on the U14-NTD dimer. The residues constituting each cluster are represented by stick models. (A) Negatively charged residues around the β-hairpins. Note that pairs of residues from the two monomers are shown. (B) Negatively charged residues on the SD4. (C) Negatively charged residues on the SD3. (D) Positively charged residues on the SD1 and SD2.
(TIF)

S4 Fig Dimensions of the groove between two U14-NTD monomers. (A) The view from the groove path. The dotted line indicated the section shown in (B). (B) Bird’s-eye view of the groove. The gray area represents the cross-section of U14-NTD molecules at the position shown in (B).
(TIF)

S5 Fig Multiple sequence alignment of HHV-6B U14, HHV-6A U14, HHV-7 U14, and HCMV UL25. The colors and symbols are the same as described in the Fig 6. Accession numbers for the sequences are as follows: HHV-6B U14: gi|4996002, HHV-6A U14:gi|9628315, HHV-7 U14: gi|1139615, and HCMV UL25: gi|822886826.
(TIF)

S6 Fig β hairpin of U14 is involved in the interaction with U11. HEK-293T cell was co-transfected with pCAGGS/U11 + pCAGGS/U14 (WT), pCAGGS/U11 + pCAGGS/U14_Δ424–426 (Δ424–426), pCAGGS/U11 + pCAGGS/U14_I414A (I414A), or pCAGGS/U11 + pCAGGS empty vector (Empty). Cells were harvested at 48 h post transfection, and lysed with TNE buffer (10 mM TrisHCl pH 7.4, 150 mM NaCl, 1 mM EDTA, and 1% Nonidet P-40). The lysate was subjected to the immunoprecipitation (IP) with anti-U14 antibody. The coprecipitates were analyzed by Western blotting (WB) with anti-U14, anti-p53, and anti-U11 antibodies. For the sake of referring to the HHV-6B U14-NTD structure, the amino acid numbering shown here is according to HHV-6B U14, although U11 and U14 in this experiment were derived from HHV-6A strain U1102, in which the residue numbering of U14 is shifted by +5 (Fig 6). Note that all of the residues examined in this experiment were identical between HHV-6A U14 and HHV-6B U14.
(TIF)
