# Supplementary material for: Crystal Structure of Human Herpesvirus 6B Tegument Protein U14
Source: PLoS Pathog. 2016 May 6;12(5):e1005594. doi: 10.1371/journal.ppat.1005594 (PMC4859480; doi:10.1371/journal.ppat.1005594)
Supplement: S1 Table — (DOCX) [file ppat.1005594.s001.docx]

**S1 Table.** **Summary of the hydrogen bonds at the dimer interface**

| Interaction site^a^ | Molecule 1 | | | Molecule 2 | | |
| --- | --- | --- | --- | --- | --- | --- |
|  | Subdomain | Residue^b^ | Group | Subdomain | Residue^b^ | Group |
| Site a | SD4 | D365 (η2) | Oδ1 | SD3 | S150 (α7) | Oγ |
|  | SD4 | D365 (η2) | Oδ2 | SD3 | K154 (α7) | Nζ |
|  | SD4 | E367 (η2–η3) | O | SD3 | K151 (α7) | Nζ |
|  | SD4 | Y368 (η2–η3) | O | SD3 | H143 (α6-α7) | Nε2 |
|  | SD4 | E370 (η2–η3) | O | SD3 | N145 (α6-α7) | Nδ2 |
| Site b | SD4 | Q376 (η3) | O | SD2 | R254 (α10) | Nη1 |
|  | SD4 | D378 (η3–η4) | O | SD2 | R254 (α10) | Nη1 |
|  | SD4 | R381 (η3–η4) | O | SD2 | R254 (α10) | Nη2 |
|  | SD4 | D383 (η3–η4) | Oδ1, Oδ2 | SD2 | R255 (α10) | Nη1, Nε |
|  | SD4 | D388 (η4–η5) | Oδ2 | SD2 | R255 (α10) | Nη1 |
|  | SD4 | D390 (η4–η5) | Oδ2 | SD1 | Q57 (α3) | Nε2 |
| Site c | SD2 | R75 (α4) | Nη2 | SD1 | E60 (α3) | O |
|  | SD2 | R75 (α4) | Nη2 | SD1 | Q61 (α3) | O |
|  | SD2 | D76 (α4) | Oδ2 | SD2 | R83 (α4) | Nη2 |
|  | SD4 | S332 (α13) | Oγ | SD2 | W259 (α19) | Nε1 |
| Site d | SD4 | T413 (β1) | O | SD2 | N296 (α11) | Nδ2 |
|  | SD4 | N419 (β1-β2) | Nδ2 | SD4 | I403 (η5-β1) | O |
|  | SD4 | N419 (β2) | O | SD4 | A404 (η5-β1) | N |
|  | SD4 | I421 (β2) | N | SD4 | I405 (η5-β1) | O |

^a^Only one of the two symmetrical interaction sites were listed here, thus the total number of hydrogen bonds is twice in the homodimer.

^b^The locations in secondary structure diagram were shown in the parentheses.
