# Supplementary material for: Crystal Structure of Human Herpesvirus 6B Tegument Protein U14
Source: PLoS Pathog. 2016 May 6;12(5):e1005594. doi: 10.1371/journal.ppat.1005594 (PMC4859480; doi:10.1371/journal.ppat.1005594)
Supplement: S2 Table — (DOCX) [file ppat.1005594.s002.docx]

**S2 Table. Propensity in the amino acid composition of U14 and homolog proteins**

| Analyzed sequence | Rate of polar, charged or Gly residues (%)^a^ | Rate of Ser residues (%) |
| --- | --- | --- |
| HHV-6B U14 |  |  |
| NTD (1-458) | 55.2 | 7.0 |
| C-terminal region (459-605) | 72.1 | 20.4 |
| HHV-6A U14 |  |  |
| N-terminal region (1-463) | 55.5 | 6.7 |
| C-terminal region (464-611) | 74.0 | 21.2 |
| HHV-7 U14 |  |  |
| N-terminal region (1-458) | 54,6 | 7.6 |
| C-terminal region (459-648) | 70.0 | 21.1 |
| HCMV UL25 |  |  |
| N-terminal region (1-180) | 69.4 | 22.8 |
| C-terminal region (459-648) | 59.2 | 5.7 |
| HCMV UL35 |  |  |
| N-terminal region (1-472) | 56.4 | 4.2 |
| C-terminal region (473-643) | 66.9 | 23.1 |

^a^This group contains following residues: Cys, Asp, Glu, Gly, His, Lys, Asn, Gln, Arg, Ser, Thr and Tyr.
