# Supplementary figures and images for: Crystal Structure of Human Herpesvirus 6B Tegument Protein U14
Source: PLoS Pathog. 2016 May 6;12(5):e1005594. doi: 10.1371/journal.ppat.1005594 (PMC4859480; doi:10.1371/journal.ppat.1005594)

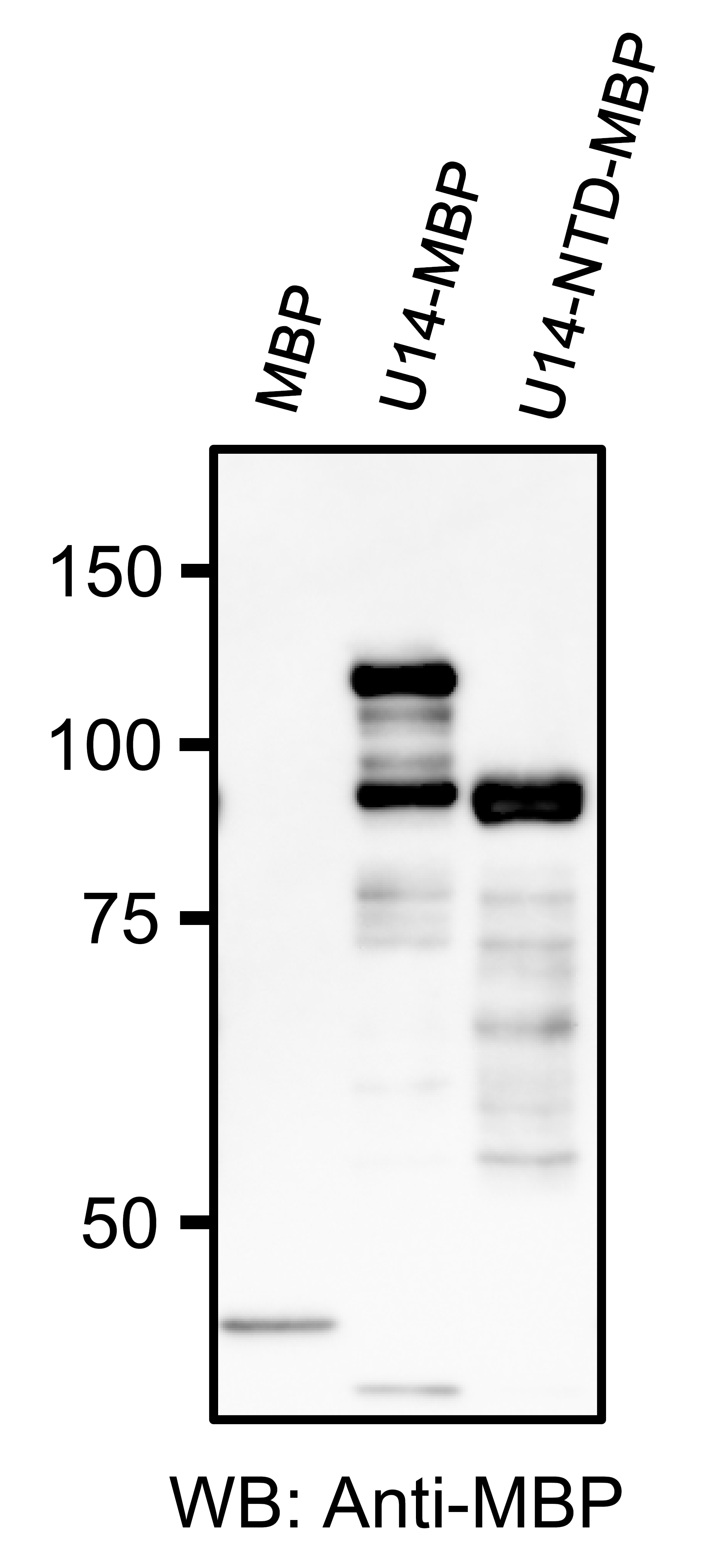

Supplement: S1 Fig — U14-MBP expressed in E. coli was degraded into multiple bands. In contrast, U14-NTD-MBP was not degraded, and the size was similar to the degraded product of U14-MBP. MBP was detected by Western blotting technique with anti-MBP antibody. (TIF) [file ppat.1005594.s003.tif]

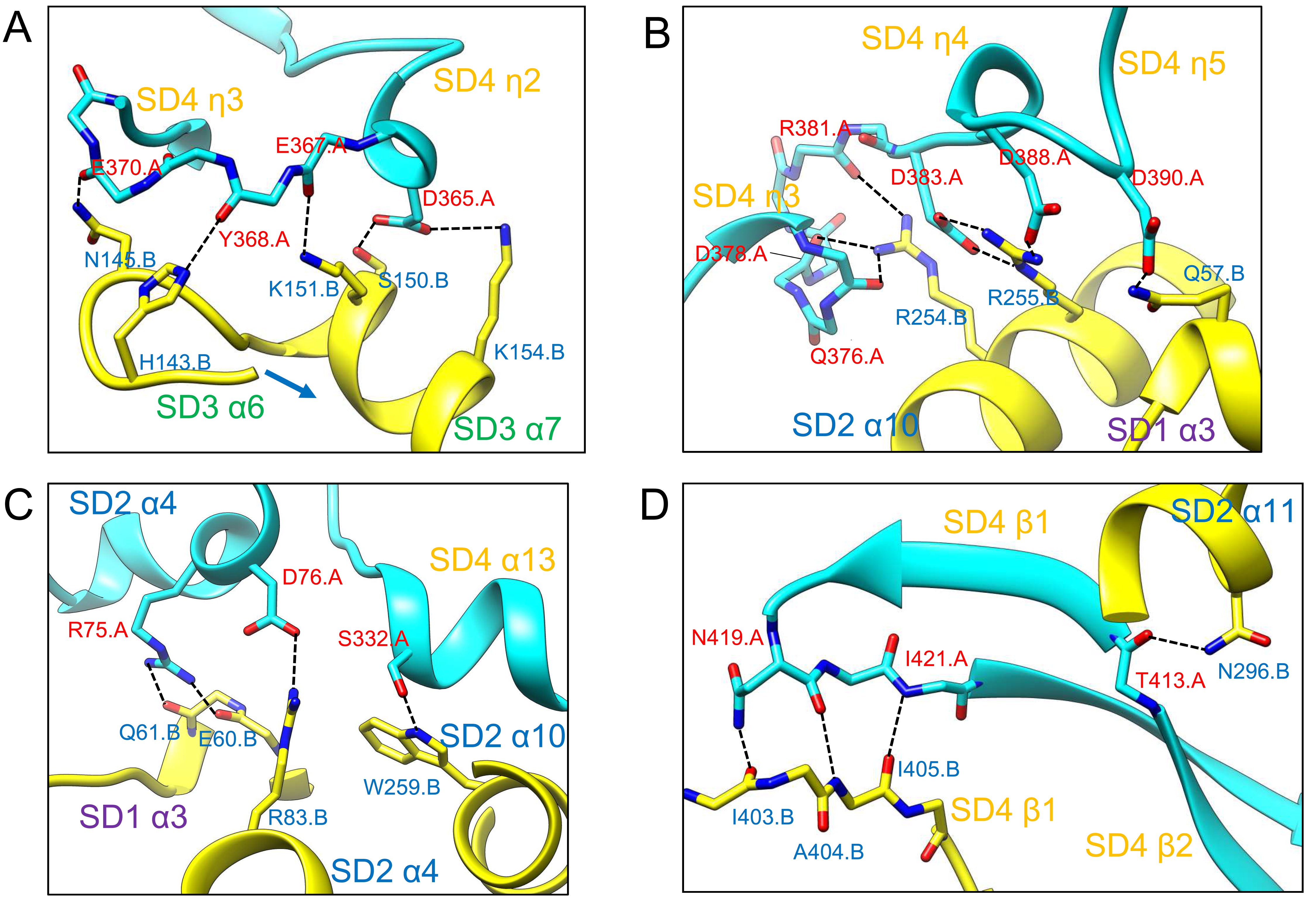

Supplement: S2 Fig — Monomers are indicated as Mol.1 (cyan) and Mol.2 (yellow). (A) Site a. The η3 and the following loop in SD4, which is located at the outermost region of the long axis, face the N-terminal region of α5 and the preceding loop in SD3 of Mol.2. At the loop region in SD4, the main-chain forms hydrogen bonds with the side-chains of N145, H143, and K151 in SD3 of Mol.2. The beta carboxyl group of D365 on the η3 forms hydrogen bonds with the side-chains of S150 and K154 of Mol.2. (B) Site b. The loop region 376–390 including η4 faces α10 and α3 of the partner molecule. The carbonyl oxygen of Q376, D378, and R381 forms hydrogen bonds with R254 of Mol.2. Three aligned aspartic acids, D383, D388, and D390 protrude from the interface and form hydrogen bonds. The side-chains of D383 and D388 form hydrogen bonds with the side-chain of R255 of Mol.2. D390 forms a hydrogen bond with Q57 in SD1 Mol.2. (C) Site c. The N-terminal tip of α4 (residues 69–79) loops back 90° and is involved in interactions with site c near the two-fold axis of the dimer. The side-chain of R75 forms hydrogen bonds with the carbonyl oxygens of E60 and Q61 in the partner molecule. D76 forms hydrogen bonds with R83 of Mol.2. This hydrogen bond is the one closest to the two-fold axis of the dimer. In the vicinity, the S332 hydroxyl group forms a hydrogen bond with the indole nitrogen of W259 in Mol.2. (D) Site d is also close to the two-fold axis, but at the opposite side of site c, around the β hairpin. At the tip of the β hairpin, the main-chain atoms of N419 and I421 form hydrogen bonds with the adjacent main-chain atoms of I405 of Mol.2. The Nδ2 group of N419 forms a hydrogen bond with the carbonyl oxygen of I403 of Mol.2. Additionally, the main-chain carbonyl oxygen of T413 is hydrogen bonded with the side-chain of N296 at α10 of Mol.2. (TIF) [file ppat.1005594.s004.tif]

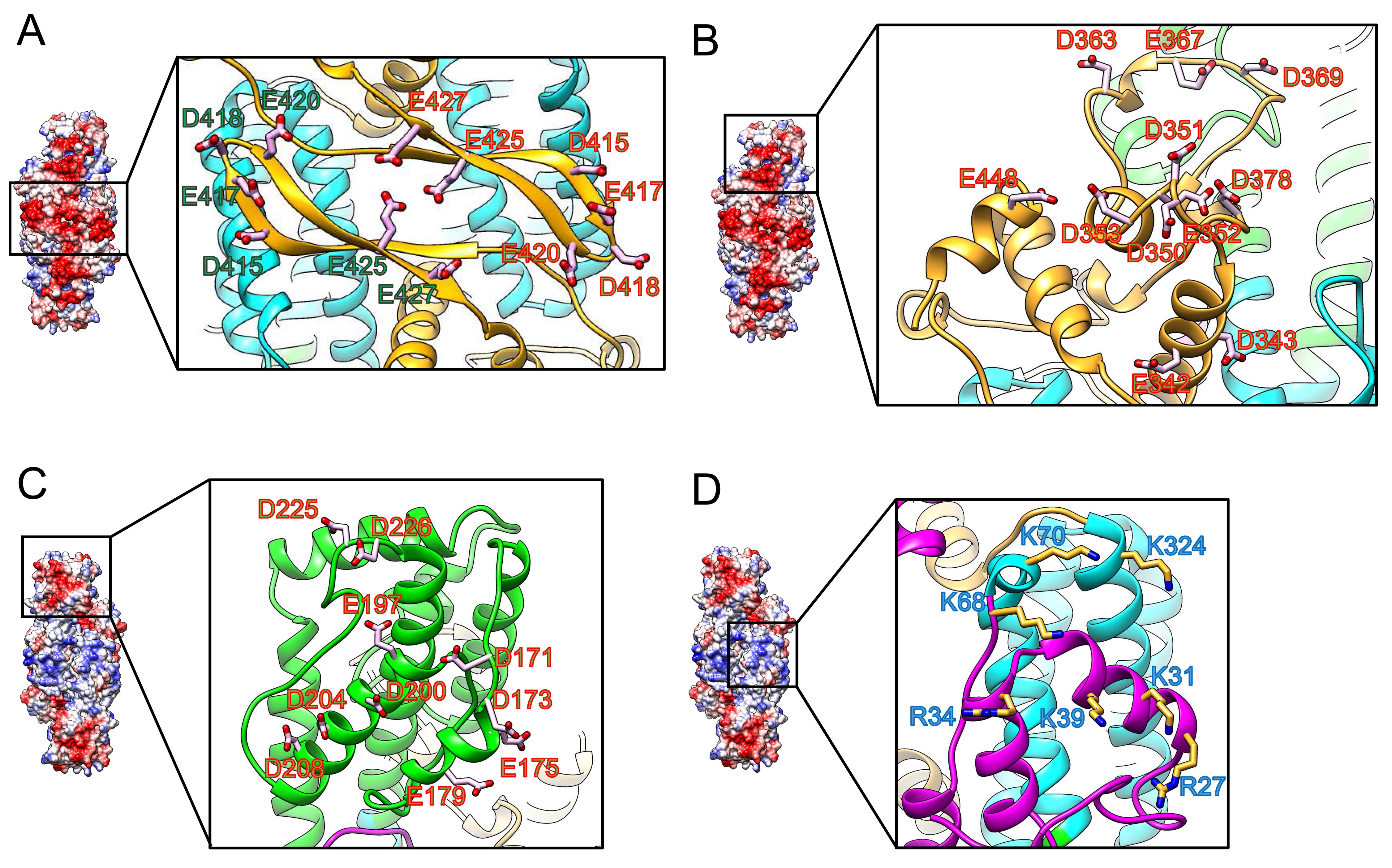

Supplement: S3 Fig — The residues constituting each cluster are represented by stick models. (A) Negatively charged residues around the β-hairpins. Note that pairs of residues from the two monomers are shown. (B) Negatively charged residues on the SD4. (C) Negatively charged residues on the SD3. (D) Positively charged residues on the SD1 and SD2. (TIF) [file ppat.1005594.s005.tif]

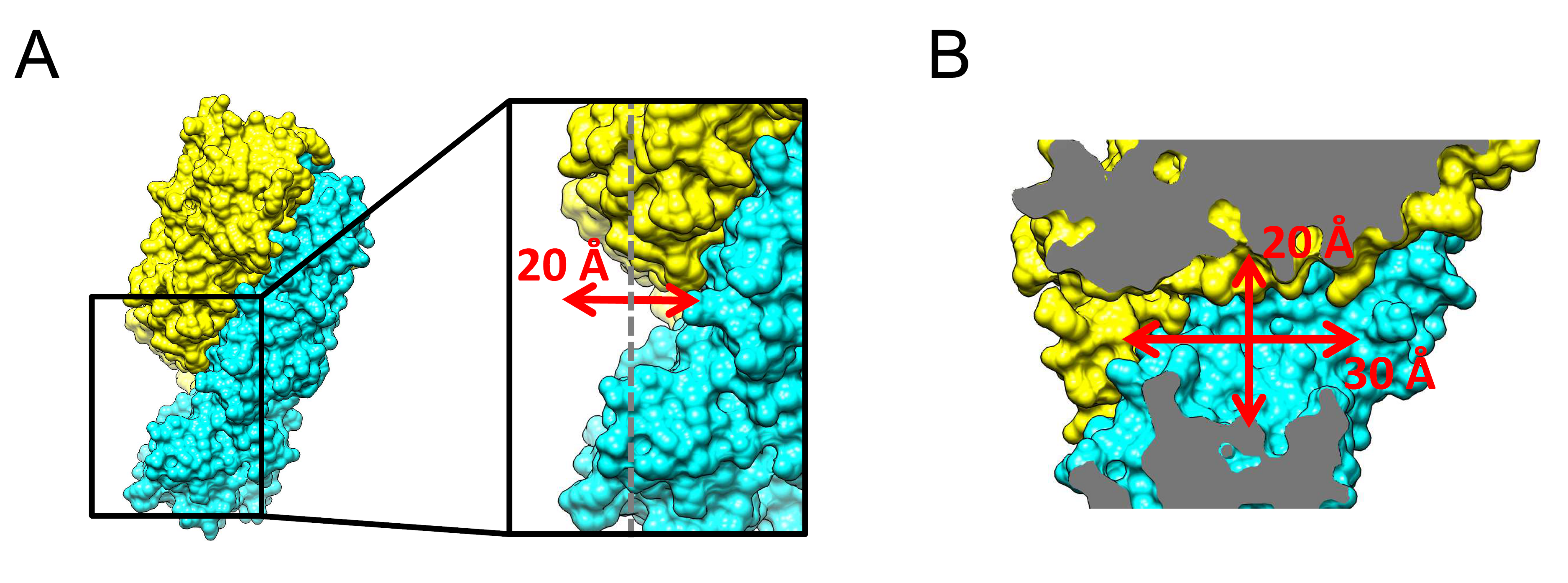

Supplement: S4 Fig — (A) The view from the groove path. The dotted line indicated the section shown in (B). (B) Bird’s-eye view of the groove. The gray area represents the cross-section of U14-NTD molecules at the position shown in (B). (TIF) [file ppat.1005594.s006.tif]

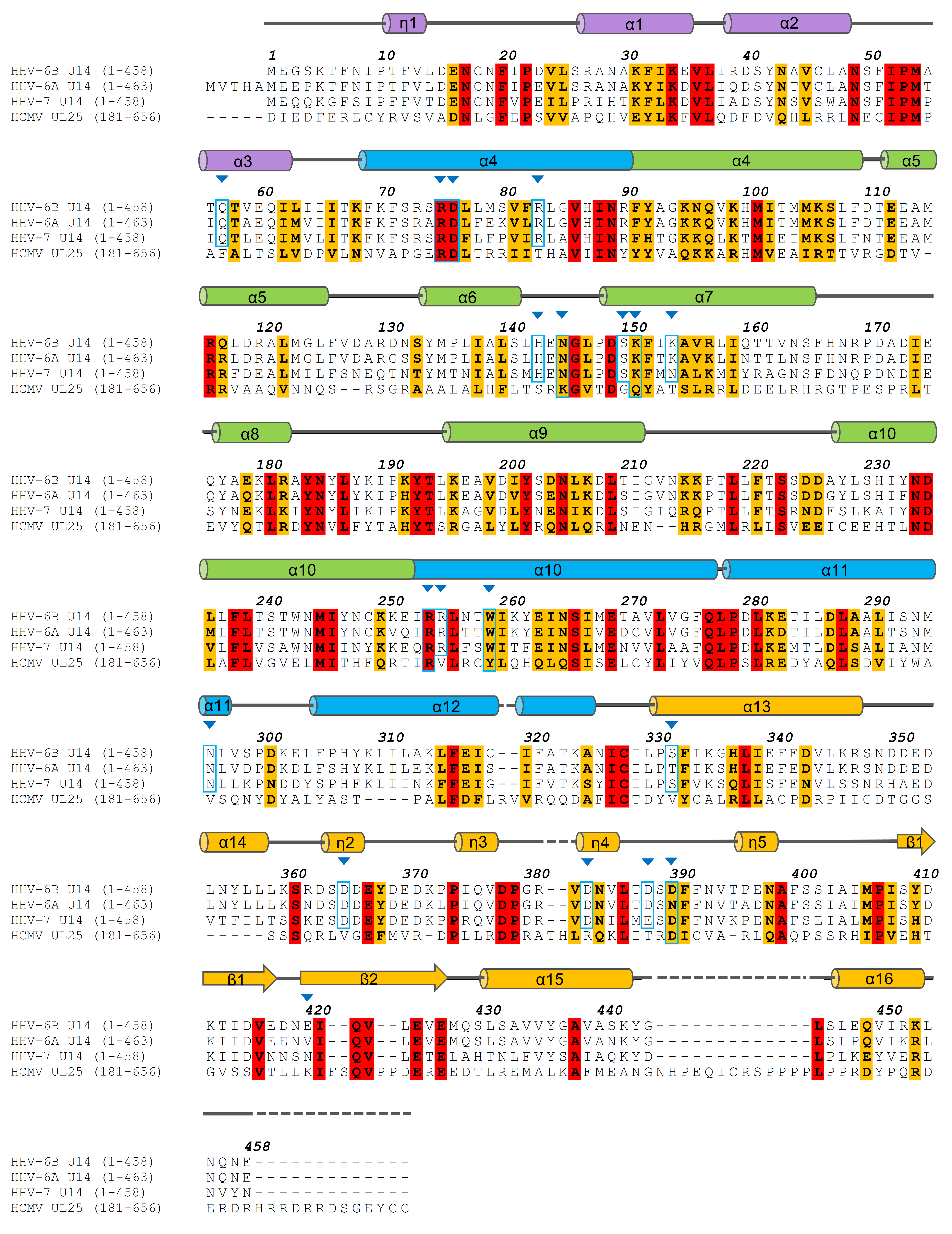

Supplement: S5 Fig — The colors and symbols are the same as described in the Fig 6. Accession numbers for the sequences are as follows: HHV-6B U14: gi|4996002, HHV-6A U14:gi|9628315, HHV-7 U14: gi|1139615, and HCMV UL25: gi|822886826. (TIF) [file ppat.1005594.s007.tif]

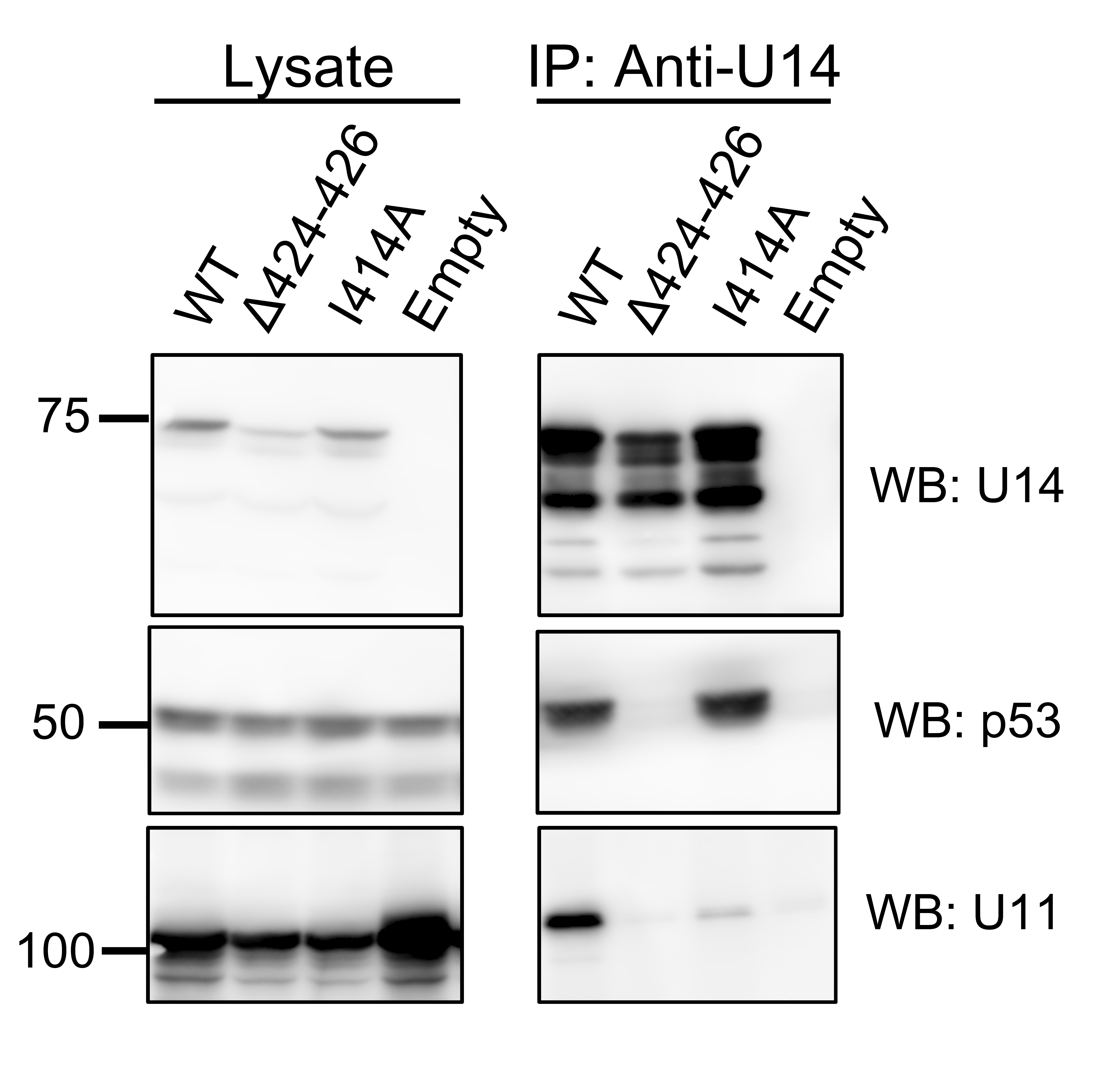

Supplement: S6 Fig — HEK-293T cell was co-transfected with pCAGGS/U11 + pCAGGS/U14 (WT), pCAGGS/U11 + pCAGGS/U14_Δ424–426 (Δ424–426), pCAGGS/U11 + pCAGGS/U14_I414A (I414A), or pCAGGS/U11 + pCAGGS empty vector (Empty). Cells were harvested at 48 h post transfection, and lysed with TNE buffer (10 mM TrisHCl pH 7.4, 150 mM NaCl, 1 mM EDTA, and 1% Nonidet P-40). The lysate was subjected to the immunoprecipitation (IP) with anti-U14 antibody. The coprecipitates were analyzed by Western blotting (WB) with anti-U14, anti-p53, and anti-U11 antibodies. For the sake of referring to the HHV-6B U14-NTD structure, the amino acid numbering shown here is according to HHV-6B U14, although U11 and U14 in this experiment were derived from HHV-6A strain U1102, in which the residue numbering of U14 is shifted by +5 (Fig 6). Note that all of the residues examined in this experiment were identical between HHV-6A U14 and HHV-6B U14. (TIF) [file ppat.1005594.s008.tif]
